# FLI1 Expression in Invasive Breast Carcinoma: Clinicopathological Correlations and Prognostic Implications

**DOI:** 10.3390/genes16111313

**Published:** 2025-11-02

**Authors:** Nusrat Jahan Doly, Dong Yeul Lee, Kazi Nafisa Tahsin, Jhuma Akhter, Shahana Sultana, Julekha Khatun, Sue-zann Chua, A. Tasleema Banu, Qingfeng Chen, Jabed Iqbal

**Affiliations:** 1 Department of Pathology, Dhaka Medical College, Dhaka 1000, Bangladesh; doctornusratjahan@gmail.com (N.J.D.); jhuma.kmc@gmail.com (J.A.); julekhacmc48@gmail.com (J.K.); 2 Division of Pathology, Singapore General Hospital Academia, Singapore 169856, Singapore; dongyeul001@gmail.com (D.Y.L.); chua.sezann@gmail.com (S.-z.C.); 3 Department of Pathology, National Institute of Burn and Plastic Surgery, Dhaka 1000, Bangladesh; kazinafisa29@gmail.com; 4 Department of Pathology, Greenlife Medical College, Dhaka 1205, Bangladesh; shahanashova935@gmail.com; 5 Translational Research and Innovation Laboratory, Singapore General Hospital Academia, Singapore 169856, Singapore; a.tasleema.banu@sgh.com.sg; 6 Institute of Molecular and Cell Biology, Agency for Science, Technology and Research, Singapore 138673, Singapore; qchen@imcb.a-star.edu.sg

**Keywords:** FLI1, breast, TME

## Abstract

Background: The E26 transformation-specific (ETS) transcription factor Friend Leukemia Integration 1 (FLI1) has been linked to breast cancer aggressiveness, stromal remodeling, and immune modulation, yet the regulatory mechanisms governing its activity remain poorly defined. Of note, various studies have shown that EWS-FLI1-mediated transcription programs are facilitated via direct recruitment and binding of the NuRD-LSD1 complex, regulating its associated gene targets. Furthermore, LSD1 inhibition exhibited reverse transcriptional profiles driven by ETS-FLI and reduced in vivo tumorigenesis in cancers. Methods: We evaluated FLI1 expression across multiple invasive breast carcinoma (IBC) cohorts to determine its prognostic significance and associations with stromal features. In parallel, we investigated FLI1 regulation in humanized breast cancer mouse models treated with an LSD1 inhibitor. Results: High FLI1 expression was associated with advanced histological grade in IBC, consistent with an oncogenic function. FLI1-high tumors also exhibited elevated stromal and immune scores, indicating a role in remodeling the tumor microenvironment. Additionally, LSD1 inhibition downregulated FLI1 target genes involving angiogenesis and invasion. Conclusions: These findings highlight the dual role of FLI1: tumor-intrinsic FLI1 promotes proliferation and invasion, whereas its transcriptional regulation in tumor and endothelial compartments likely reflects LSD1 dependence. Collectively, our results support a mechanistic model in which LSD1–FLI1 crosstalk is involved in immune and stromal remodeling, positioning FLI1 as both a marker of tumor aggressiveness and a potential predictor of response to epigenetic therapies in breast cancer.

## 1. Introduction

Triple-negative breast cancer (TNBC) remains one of the most aggressive breast cancer subtypes due to the lack of hormone receptors and human epidermal growth factor receptor 2 (HER2) amplification, which limits therapeutic options and contributes to poor prognosis [1]. Emerging evidence underscores the tumor microenvironment (TME) as a key determinant of TNBC progression and therapeutic response. Integrative strategies combining epigenetic modulators with immunotherapy are being investigated to overcome immune evasion and therapeutic resistance [2].

FLI1, an ETS transcription factor, is critical for hematopoietic and endothelial differentiation but has also been implicated in breast cancer aggressiveness [3]. Elevated FLI1 expression correlates with higher histological grade, stromal remodeling, and poor clinical outcomes in TNBC [4]. FLI1 activity is influenced by chromatin regulation, as ETS factors, including FLI1, frequently co-occupy regulatory regions with LSD1 and other chromatin remodelers, indicating a functional intersection between epigenetic repression and ETS-driven transcriptional programs [5].

Lysine-specific demethylase 1 (LSD1) is a histone demethylase that regulates H3K4 and H3K9 methylation to maintain repressive chromatin states [6]. In cancer, LSD1 suppresses interferon signaling, antigen presentation, and immune-stimulatory endogenous retroviral elements, whereas its inhibition reactivates type I interferon pathways, enhances T cell infiltration, and sensitizes tumors to immune checkpoint blockade (ICB) [7]. Mechanistically, LSD1 has been shown to co-occupy genomic loci with ETS family transcription factors, including FLI1, suggesting direct regulatory interplay in transcriptional programs relevant to tumor progression [8,9].

Given the central role of FLI1 in cellular regulation, understanding its expression patterns in breast cancer and its contributions to tumor progression and metastasis is critical. This study aims to investigate FLI1 expression in invasive breast carcinoma (IBC) and evaluate its association with histopathological grading. Specifically, it seeks to determine whether FLI1 expression correlates with aggressive tumor characteristics and clinical outcomes, thereby providing insights into its potential as a prognostic marker. The study addresses the following research objectives: (a) the expression pattern of FLI1 in IBC, (b) the association of FLI1 expression with histopathological grading and prognosis in IBC, and (c) the influence of FLI1 on the tumor microenvironment in IBC. To address these objectives, we employed a comprehensive approach integrating immunohistochemical analysis of FLI1 expression in a well-annotated cohort of invasive breast carcinoma cases, correlation with clinicopathological parameters including histological grade and survival outcomes, and profiling of immune and stromal components within the tumor microenvironment.

## 2. Materials and Methods

### 2.1. Study Design and Population

A cross-sectional observational study was carried out on female patients diagnosed with invasive breast carcinoma (IBC) through histopathological examination at the Department of Pathology, Dhaka Medical College, between September 2022 and August 2024. A total of 100 IBC cases, obtained from wide local excision and mastectomy specimens, were included in the study. Immunohistochemical analysis was performed on 60 of these cases. The study protocol was approved by the Institutional Review Board of Dhaka Medical College. Informed consent was obtained from all participants, and confidentiality of their information was maintained.

### 2.2. List of Variables Examined

Demographic variables (age), pathological characteristics (laterality, tumor type, site, size, focality, presence of in situ component, lymphovascular invasion, lymph node involvement, microcalcification, necrosis, hormonal status, tumor grade, pT stage, and pN stage), and FLI1 expression parameters (percentage of positively stained tumor area, staining intensity, and overall expression level) were recorded. Patient demographic and clinical information was recorded using a structured proforma. Informed written consent was obtained, along with relevant clinical history.

### 2.3. Selection Criteria

This study enrolled female patients of any age, including all mastectomy and wide local excision specimens diagnosed with invasive breast carcinoma, with available immunohistochemical reports for receptor status (ER, PR, HER2). Exclusion criteria included patients who received neoadjuvant chemotherapy or radiotherapy, cases of recurrent breast carcinoma, HER2-equivocal cases, and those with incomplete clinical or pathological information.

### 2.4. Histopathological Evaluation

Gross examination documented tumor size and axillary nodal status. Paraffin-embedded sections were stained with hematoxylin and eosin (H&E) and evaluated for histologic subtype, grade, staging, and lymphovascular invasion.

### 2.5. Immunohistochemical Procedure

Paraffin-embedded sections (3–4 µm thick) were immunostained using a standard labeled streptavidin–biotin system (Genemed, South San Francisco, CA, USA) with Anti-FLI-1 polyclonal antibody (Chongqing Biospes Co., Ltd., Chongqing, China) at a 1:50 dilution. Sections were deparaffinized, followed by antigen retrieval in 10 mmol/L citrate monohydrate buffer (pH 6.0) heated in a microwave for 20 min. Diaminobenzidine (DAB) served as the chromogen, and immunodetection was performed using commercial detection kits (Dako, Glostrup, Denmark).

### 2.6. Immunohistochemical Analysis

Cells were considered immunohistochemically positive for FLI1 when a brownish nuclear staining was observed in tumor cell nuclei [10]. To allow for a semi-quantitative and reproducible assessment, immunohistochemical staining was scored on a scale of 0–9, based on the percentage of positive tumor cells and staining intensity (Table 1 and Table 2). The FLI1 score was calculated by multiplying the staining intensity by the percentage of positive cells, yielding possible scores of 0, 1, 2, 3, 4, 6, and 9. A cutoff score of 4 was used to define FLI1 expression: scores ≥ 4 indicated high expression, while scores ≤ 3 indicated low expression. Normal tonsillar tissue was used as an external positive control for the staining procedure.

### 2.7. Follow-Up and Statistical Analysis

Follow-up data were obtained from electronic medical records. Survival outcomes were estimated with Kaplan–Meier analysis and compared between subgroups with log-rank statistics. Multivariate Cox Regression was carried out to evaluate the effect of FLI1 tumor cell expression level, with survival adjusted for the effects of age, grade, tumor size, lymph node stage, and lymph node positivity. Descriptive statistics, Chi-square tests, and Fisher’s exact tests were performed, with a *p*-value < 0.05 considered statistically significant.

### 2.8. Database

Data were obtained from multiple publicly available breast cancer genomic resources. Information from the Molecular Taxonomy of Breast Cancer International Consortium (METABRIC) and the Cancer Genome Atlas (TCGA) was accessed via cBioPortal (https://www.cbioportal.org/, accessed on 24 September 2022) [11,12]. Data from the Fudan University Shanghai Cancer Center (FUSCC) were obtained from BioSino (https://www.biosino.org/node/, Project ID: OEP00015, accessed on 24 September 2022) [12] and the Fudan Data Portal for Cancer Genomics (https://doi.org/10.6084/m9.figshare.19783498.v5, accessed on 24 September 2022) [13,14]. For invasive breast carcinoma (IBC) cohorts, the case numbers were METABRIC (*n* = 1886), FUSCC (*n* = 343), and TCGA (*n* = 816). Mass spectrometry-based proteogenomic data of for IBC samples were also obtained from cBioPortal [11,12,15]. The ESTIMATE (Estimation of Stromal and Immune cells in Malignant Tumor tissues using Expression data) algorithm was applied to calculate stromal scores (reflecting stromal content) and immune scores (reflecting immune cell infiltration), which were compared between FLI1-high and FLI1-low samples.

### 2.9. Humanized Breast Cancer Mouse Model

Humanized immune system (HIS) mice were transplanted with 1 × 10^6^ MDA-MB-231 cells. Tumor volumes were monitored twice weekly until reaching approximately 80–100 mm^3^, at which point mice were treated intraperitoneally with the LSD1 inhibitor phenelzine (40 mg/kg daily) [16]. All injections were performed under sterile conditions, and mice were monitored daily for treatment-related effects throughout the study. Tumors from the endpoint were harvested and submitted for bulk-RNA sequencing. Raw counts were subjected to differential gene expression analysis using DESeq2, with fold change estimation values (between treatment group and control group) used for downstream analysis.

## 3. Results

### 3.1. Demographics

The mean age of patients was 46.9 years, with the highest frequency observed in the 46–55-year age group (Table 3), consistent with previous studies reporting peak breast cancer incidence in late middle age.

### 3.2. Tumor Type and Stage

Infiltrating duct carcinoma (IDC) was the predominant tumor type, accounting for 93.0% of cases (Table 4). Representative images of IDC (grades 2–3) and invasive lobular carcinoma (grades 1–2) with varying FLI1 immunostaining intensity from low to high are provided in the supplementary material. Most tumors were classified as pT2 (57.0%) (Table 5), with sizes ranging from 2.1 to 5.0 cm (Table 6). Lymphovascular invasion (LVI) was observed in 44.0% of cases (Table 7), possibly due to differences in sampling.

### 3.3. Distribution of Nodal Involvement and Hormonal Receptor Status in Study Cohort

Nodal metastasis was present in 42.0% of cases, whereas 52.0% showed no nodal involvement. Hormonal receptor analysis revealed estrogen receptor (ER) positivity in 60.0% of cases and progesterone receptor (PR) positivity in 45.0%, with a minority of HER2-enriched tumors (6.0%) (Table 8).

### 3.4. Histological Grading and Its Association with FLI1 Expression

**Figure 1:**
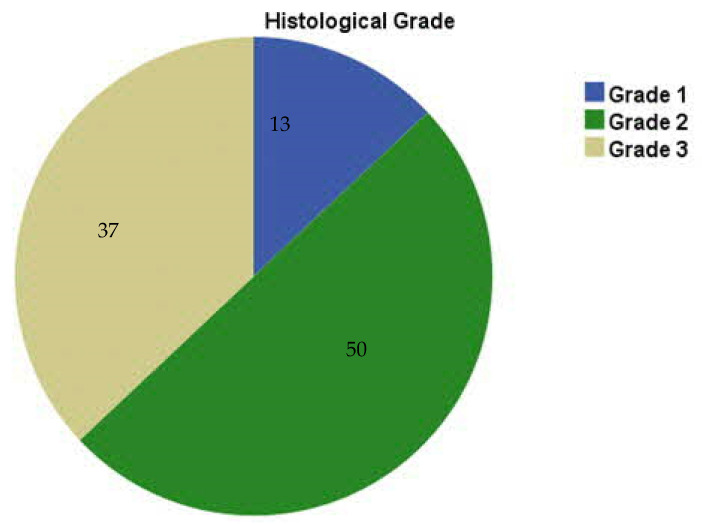
Distribution of the study cases according to histological grade (n = 100).

Within the study cohort, Grade 2 tumors were the most prevalent (50.0%), followed by Grade 3 (37.0%) and Grade 1 (13.0%) (Figure 1), consistent with previous studies [17,18,19].

A significant correlation was observed between elevated FLI1 expression and higher histological grade (*p* < 0.001), with 60% of Grade 3 tumors exhibiting high FLI1 levels (Table 9). Although not reaching statistical significance, cases with high FLI1 expression tended to demonstrate increased nodal involvement.

### 3.5. Kaplan–Meier Survival and Stromal/TIL Analyses

**Figure 2:**
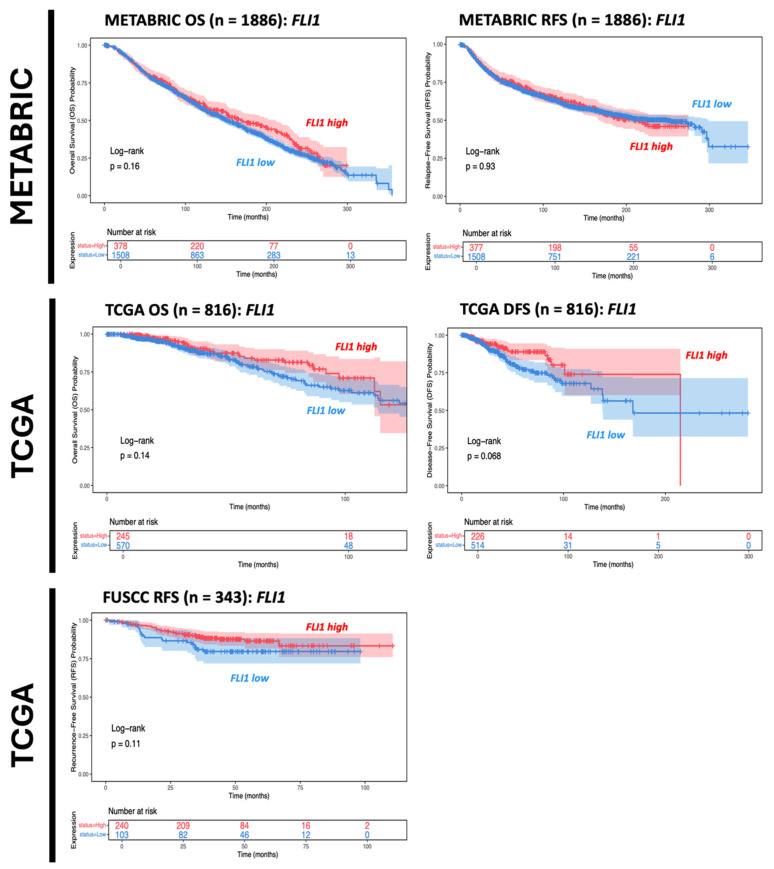
Kaplan–Meier survival analysis comparing data from different databases for IBC cohort cases. Red line indicates FLI-high population of IBC patients; Blue line indicates FLI-low population of IBC patients.

**Figure 3:**
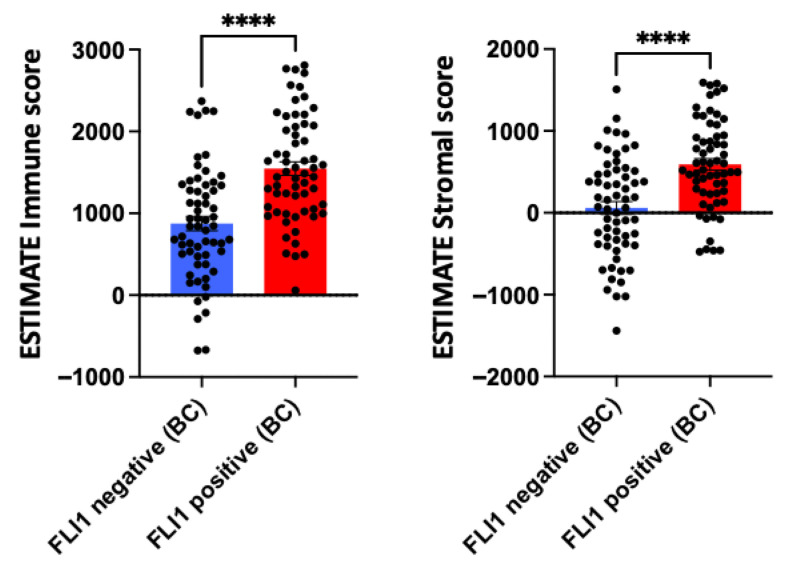
ESTIMATE immune and stromal scores for invasive breast carcinoma (IBC) between FLI1-negative and FLI1-positive cases. Data are presented as mean ± SEM, statistical significance was determined by unpaired *t*-test; **** *p* < 0.0001.

Within IBC cohorts, *FLI1* mRNA expression levels were shown to not be significantly associated with survival outcomes (Figure 2). This is concordant with existing studies with conflicting results over the prognostic function of FLI1 in breast cancer [20]. Interestingly, FLI1 expression in breast cancer samples was positively associated with both immune and stromal infiltration, underscoring FLI1’s role in tumor stroma and immune infiltration (Figure 3) [3].

### 3.6. FLI1-LSD1 Axis in Oncogenesis-Associated Transcriptional Programs

**Figure 4:**
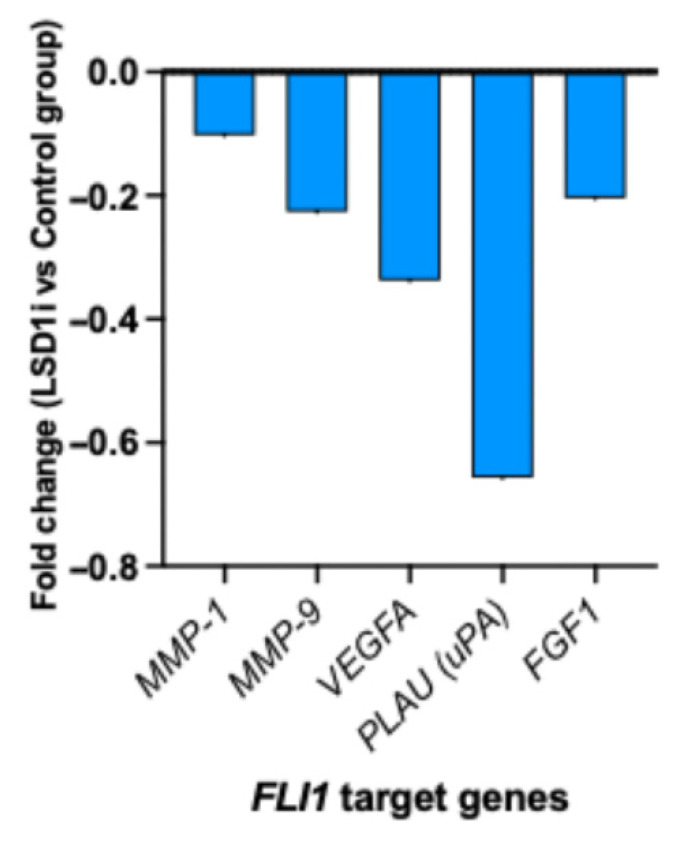
LSD1 inhibition and transcriptional regulation of FLI1 target genes in breast cancer mouse model.

In a humanized breast cancer mouse model, LSD1 inhibition resulted in reduced FLI1 target gene signatures related to angiogenesis (VEGFA, FGF-1, MMP-1, MMP-9), migration, and invasion (PLAU) (Figure 4).

## 4. Discussion

This study characterized FLI1 protein expression in invasive breast carcinoma and assessed its association with histopathological grade, clinicopathological features, and patient survival. The results indicate that FLI1 may play a role in tumor progression, aggressiveness, and prognosis. Additionally, we explored the modulation of FLI1 in breast cancer under LSD1 inhibition. Using breast cancer mouse models, LSD1 inhibition influenced FLI1 target genes related to tumor progression. These findings suggest that LSD1 inhibition may regulate tumor and endothelial programs regulated by FLI1.

Kaplan–Meier analyses revealed variable associations between FLI1 expression and clinical outcomes across datasets. While higher FLI1 immunostaining correlated with higher histological grading, FLI1 mRNA levels did not present a significant association with prognostic trends across invasive breast cancer cohort samples. These observations suggest that the prognostic value of FLI1 may be context-dependent, influenced by factors such as tumor subtype, genetic background, and treatment strategies. Our findings align with prior studies linking FLI1 expression to higher histological grade, consistent with the role of ETS factors in regulating proliferation, differentiation, and apoptosis in breast cancer [6].

No significant correlation was observed between FLI1 expression and pathological T or N stage (*p* = 0.604 and 0.125, respectively), in contrast to Song et al. (2015), who reported an association between FLI1 score and lymph node metastasis, potentially reflecting differences in sample characteristics [7].

FLI1 expression was not significantly associated with ER, PR, or HER2 status, suggesting that its role in breast cancer progression may be independent of these canonical molecular markers and could serve as a complementary biomarker. This contrasts with Lafta (2017), who reported reduced FLI1 expression across all stages of primary breast carcinoma and correlations with ER, PR, and HER2 [8].

FLI1 positivity appeared to influence immune and stromal infiltration in breast cancer [4]. FLI1-positive tumors displayed significantly higher stromal scores, indicating greater abundance of stromal components such as fibroblasts and enriched extracellular matrix and vasculature. This enrichment may reflect a complex tumor microenvironment, potentially contributing to immune modulation, therapeutic resistance, and tumor progression. The association between FLI1 and stromal content suggests FLI1’s role in stromal remodeling or fibroblast activation, warranting further mechanistic investigation.

Given that ETS-FLI transcriptional activity can be regulated by LSD1 in other cancers, our results similarly demonstrated that LSD1 inhibition could repress FLI target genes related to angiogenesis and migration, thereby promoting cancer progression or higher histological grading [21]. Overall, our results suggest the role of the FLI1-LSD1 axis in the induction of tumor migration, angiogenesis, and tumor progression through direct regulation of ECM-degrading proteases in cancer and stromal cells [4,22,23]. However, given reports of tumor-intrinsic FLI1 promoting proliferation and invasion [6], some of this signal may represent adaptive responses to epigenetic stress. These dual roles underscore the complexity of FLI1 biology in TNBC. These findings suggest two translational opportunities: (i) FLI1 could be used as a pharmacodynamic biomarker of breast cancer metastastatic potential, capturing stromal and immune remodeling, and (ii) combining direct FLI1 inhibition and LSD1 blockade may yield stnergistic therapeutic effects. Single-cell and spatial transcriptomic analyses will be critical to delineate the cellular origin of FLI1 and determine whether its elevation predominantly reflects immune activation or malignant adaptation.

This study has several limitations: (a) The assessment of lymphovascular invasion was based solely on histopathology, potentially introducing bias. (b) Pathological staging included only tumor size (pT) and lymph node status (pN), excluding metastasis (pM). (c) Immunohistochemistry was performed in a limited subset of 60 cases. (d) ER, PR, and HER2 data were obtained from various laboratory reports, and molecular subtyping was not conducted. (e) Long-term patient follow-up data were unavailable, precluding outcome analyses. (f) Newly defined subtypes based on immune and stromal infiltration were not externally validated. And lastly, (g) not all histological subtypes of invasive breast carcinoma were represented in this study.

## 5. Conclusions

This study highlights the potential role of FLI1 in promoting tumor progression and stromal remodeling in invasive breast carcinoma. Higher FLI1 protein expression was associated with increased histological grades and enriched stromal features, suggesting a role in tumor aggressiveness and microenvironment modulation. Although FLI1 expression was not correlated with patient survival, its modulation under LSD1 inhibition points to a regulatory axis with therapeutic relevance. Collectively, these findings support the consideration of FLI1 as a context-dependent biomarker for breast cancer and a potential target in epigenetic therapy strategies. Limitations such as a limited sample size, lack of molecular subtyping, and absence of longitudinal outcome data underscore the need for further validation in larger, well-characterized cohorts. Future studies leveraging multi-omics will be essential to disentangle the tumor-intrinsic and microenvironmental contributions of FLI1 expression.

## Figures and Tables

**Table 1:** Scoring grade for positive tumor cells.

Percentage (%) of Tumor Cells	Scoring
No positive tumor cells	0
Less than 10% positive tumor cells	1
10–50% positive cells	2
More than 50% positive cells	3

**Table 2:** Staining intensity classification for tumor cells.

Staining	Scoring
No staining	0
Weak staining	1
Moderate staining	2
Strong staining	3

**Table 3:** Distribution of the study cases according to age (n = 100).

Age	Frequency	Percentage
26–35	17	17.0
36–45	31	31.0
46–55	34	34.0
56–65	13	13.0
>66	5	5.0
Total	100	100.0
Mean ± SD (min-max)	46.94 ± 10.67 (26–74)	

**Table 4:** Distribution of study cases according to type of tumor (n = 100).

Type of the Tumor	Frequency	Percentage
Infiltrating duct carcinoma	93	93.0
Invasive lobular carcinoma	4	4.0
Invasive carcinoma with mixed ductal and lobular features	2	2.0
Mucinous carcinoma	1	1.0

**Table 5:** Distribution of the study cases according to pathological T stage (n = 100).

Pathological T Stage	Frequency	Percentage
pT1b	2	2.0
pT1c	15	15.0
pT2	57	57.0
pT3	13	13.0
pT4b	13	13.0

**Table 6:** Distribution of the study cases according to tumor size (n = 100).

Size of the Tumor	Frequency	Percentage
≤2 cm	16	16.0
2.1–5.0 cm	57	57.0
>5 cm	27	27.0

**Table 7:** Distribution of the study cases according to histological features (n = 100).

Histopathological Findings	Frequency	Percentage
In Situ component		
Present	56	56.0
Not identified	44	44.0
LVI		
Present	44	44.0
Not identified	56	56.0
Lymph node involvement		
Involved	42	42.0
Uninvolved	52	52.0
Cannot be assessed	6	6.0
Microcalcification		
Present	27	27.0
Not identified	73	73.0
Necrosis		
Present	58	58.0
Not identified	42	42.0

**Table 8:** Distribution of the study cases according to hormonal status (n = 100).

Hormonal Status	Frequency	Percentage
ER		
Positive	60	60.0
Negative	40	40.0
PR		
Positive	45	45.0
Negative	55	55.0
Her2		
Positive	6	6.0
Negative	94	94.0

**Table 9:** Summary of distribution of the study cases and association of FLI1 expression with different clinicopathological parameters.

Clinicopathological Parameters	FLI1 Expression (n = 60)		*p* Value
	High (n = 33)	Low (n = 27)	
Age (range 25–75 y), median ->	44.3	46.7	0.939
25–35	6 (18.2%)	4 (14.8%)	
36–45	13 (39.4%)	9 (33.3%)	
46–55	10 (30.3%)	9 (33.3%)	
56–65	3 (9.1%)	3 (11.1%)	
≥66	1 (3.0%)	2 (7.4%)	
Hormonal status (immunoprofile)	Basal like	Luminal B	
ER positive	14 (42.4%)	16 (49.3%)	0.194 Not significant
ER negative	19 (57.6%)	11 (47.0%)	
PR positive	13 (39.4%)	10 (37.0%)	0.852
PR negative	20 (60.6%)	17 (63.0%)	
Her2 positive	1 (3.0%)	2 (7.4%)	0.598 Not significant
Her2 negative	32 (97.0%)	25 (92.6%)	
Tumor size (range 0–6 cm), median ->	3	3.1	0.604
pT1	5 (15.2%)	4 (14.8%)	
pT2	21 (63.6%)	21 (77.8%)	
pT3	4 (12.1%)	1 (3.7%)	
pT4	3 (9.1%)	1 (3.7%)	
Histological grade (range 3–9), median ->	7.71	5.74	<0.001, Significant
Grade 1	0 (0.0%)	9 (33.3%)	
Grade 2	13 (39.4%)	17 (63.0%)	
Grade 3	20 (60.0%)	1 (3.7%)	
Lymph nodes (range 1–12), median ->	2.75	2.84	0.125
pN0	18 (60.0%)	12 (48.0%)	
pN1a	4 (13.3%)	4 (16.0%)	
pN2a	3 (10.0%)	8 (32.0%)	
pN3a	5 (16.7%)	1 (4.0%)	

## Data Availability

The raw data supporting the conclusions of this article will be made available by the authors on request.

## Supplementary Materials

The following supporting information can be downloaded at: https://www.mdpi.com/article/10.3390/genes16111313/s1. Figure S1: High FLI1 immunohistochemical expression in normal tonsillar tissue (40×); Figure S2: H&E staining of infiltrating duct carcinoma, Grade I (10×); Figure S3: Low FLI1 immunochemical expression in infiltrating duct carcinoma, Grade I (10×); Figure S4: H&E staining of infiltrating duct carcinoma, Grade II (10×); Figure S5: High FLI1 immunochemical expression in moderately infiltrating duct carcinoma, (10×); Figure S6: H&E staining of infiltrating duct carcinoma, Grade III (10×); Figure S7: High FLI1 immunochemical expression in poorly differentiated infiltrating duct carcinoma, (10×); Figure S8: H&E staining of invasive lobular carcinoma, Grade I (10×); Figure S9: Low FLI1 immunochemical expression in invasive lobular carcinoma, Grade I (10×).

## Abbreviations

The following abbreviations were used in this manuscript.

ETS	E26 transformation-specific
FLI1	Friend Leukemia Integration 1
LSD1	Lysine specific demethylase 1
ICB	Immune checkpoint blockade
TNBC	Triple-negative breast cancer
HER2	Human epidermal growth factor receptor 2
TME	Tumor microenvironment
IBC	Invasive breast carcinoma
H&E	Hematoxylin and eosin
DAB	Diaminobenzidine
IHC	Immunohistochemistry
METABRIC	Molecular Taxonomy of Breast Cancer International Consortium
TCGA	The Cancer Genome Atlas
FUSCC	Fudan University Shanghai Cancer Center
HIS	Humanized immune system
pT	Tumor size
pN	Lymph node involvement
pM	Excluding metastasis
ER	Estrogen receptor
PR	Progesterone receptor
IDC	Infiltrating duct carcinoma
LVI	Lymphovascular invasion
