# Supplementary material for: FLI1 Expression in Invasive Breast Carcinoma: Clinicopathological Correlations and Prognostic Implications
Source: Genes (Basel). 2025 Nov 2;16(11):1313. doi: 10.3390/genes16111313 (PMC12652689; doi:10.3390/genes16111313)
Supplement: Supplementary file 1 [file genes-16-01313-s001.zip › genes-3861084-supplementary.pptx]

## Slide 1
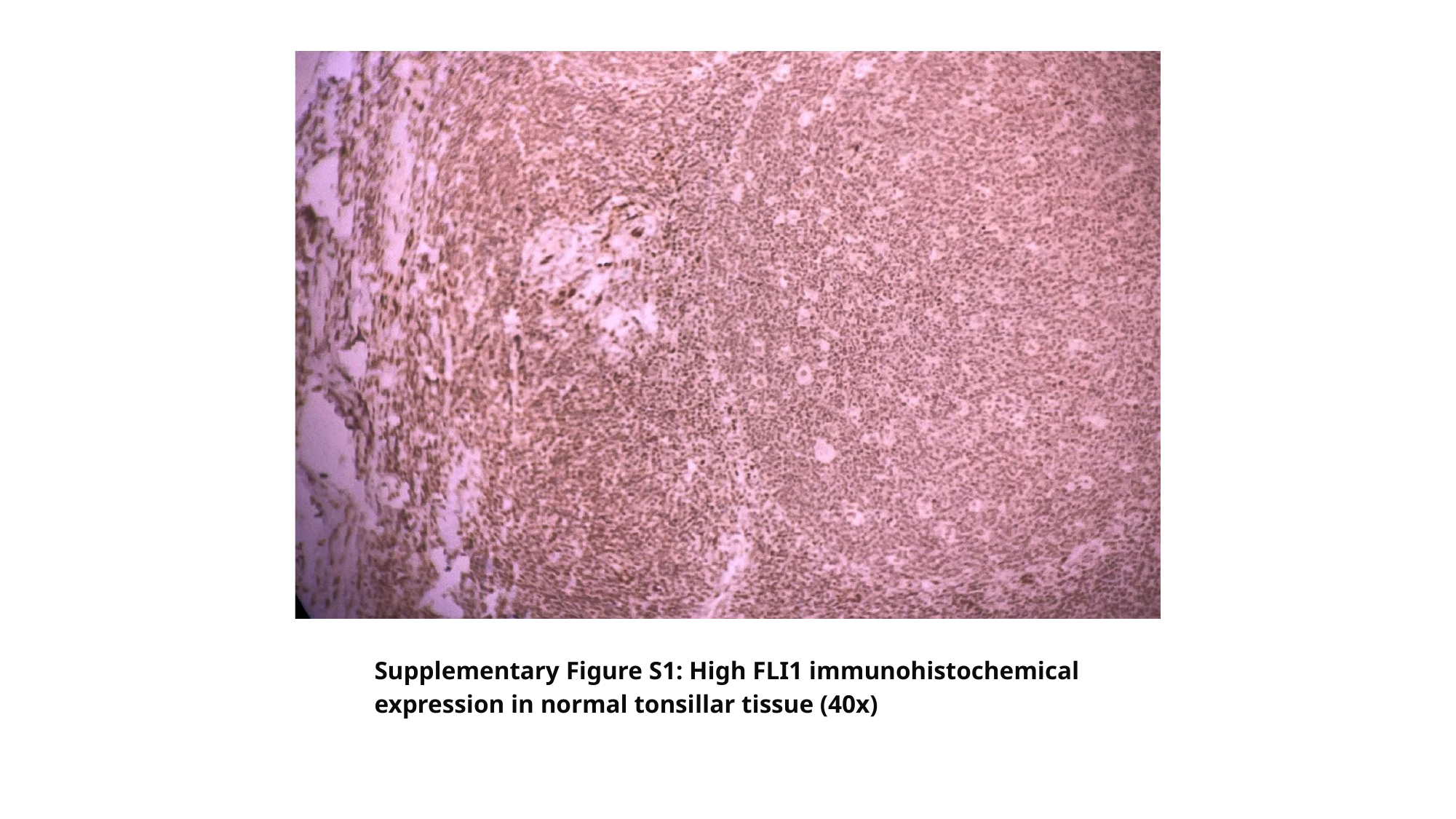

Supplementary Figure S1: High FLI1 immunohistochemical expression in normal tonsillar tissue (40x)

## Slide 2
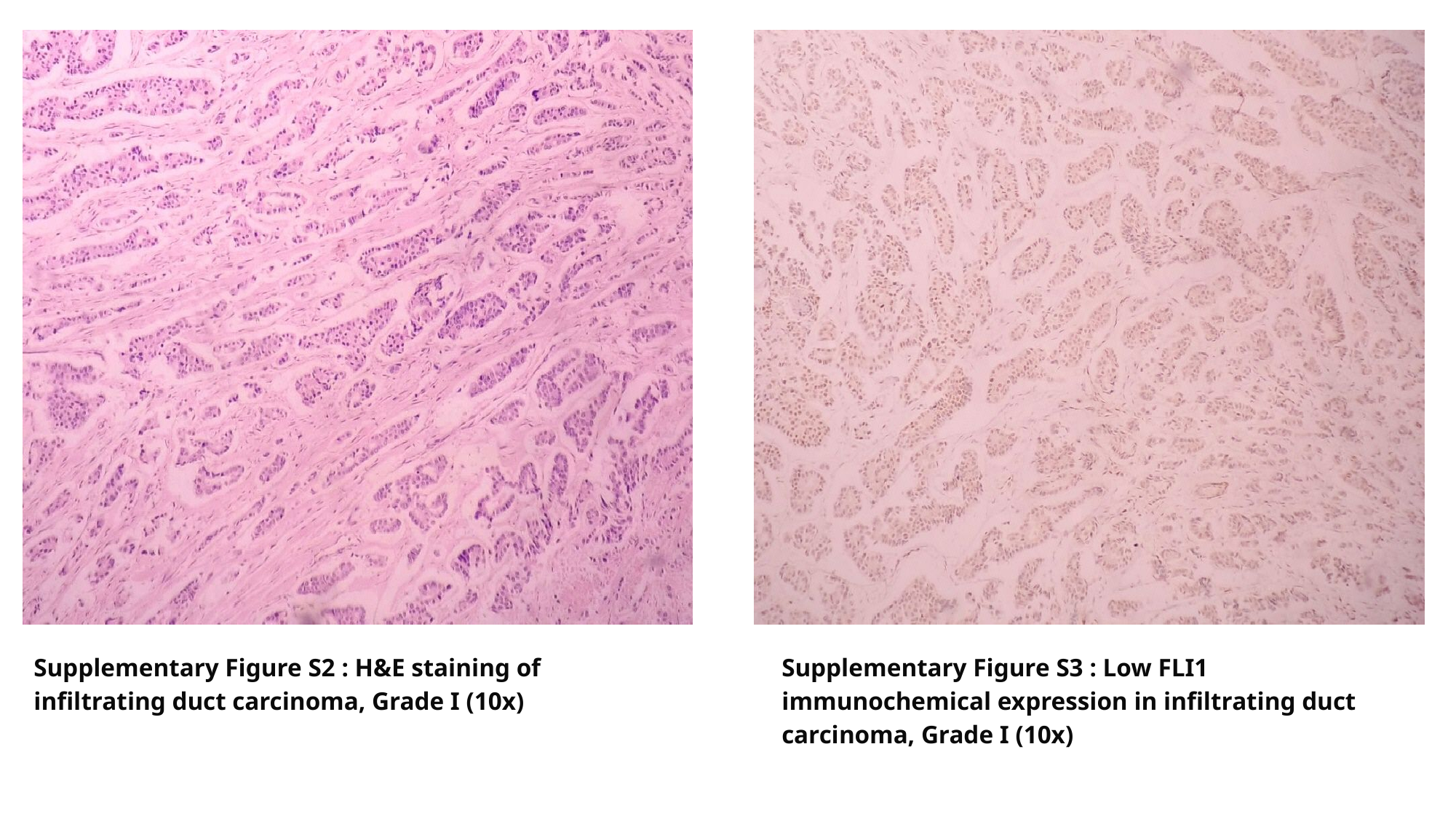

Supplementary Figure S2 : H&E staining of infiltrating duct carcinoma, Grade I (10x)
Supplementary Figure S3 : Low FLI1 immunochemical expression in infiltrating duct carcinoma, Grade I (10x)

## Slide 3
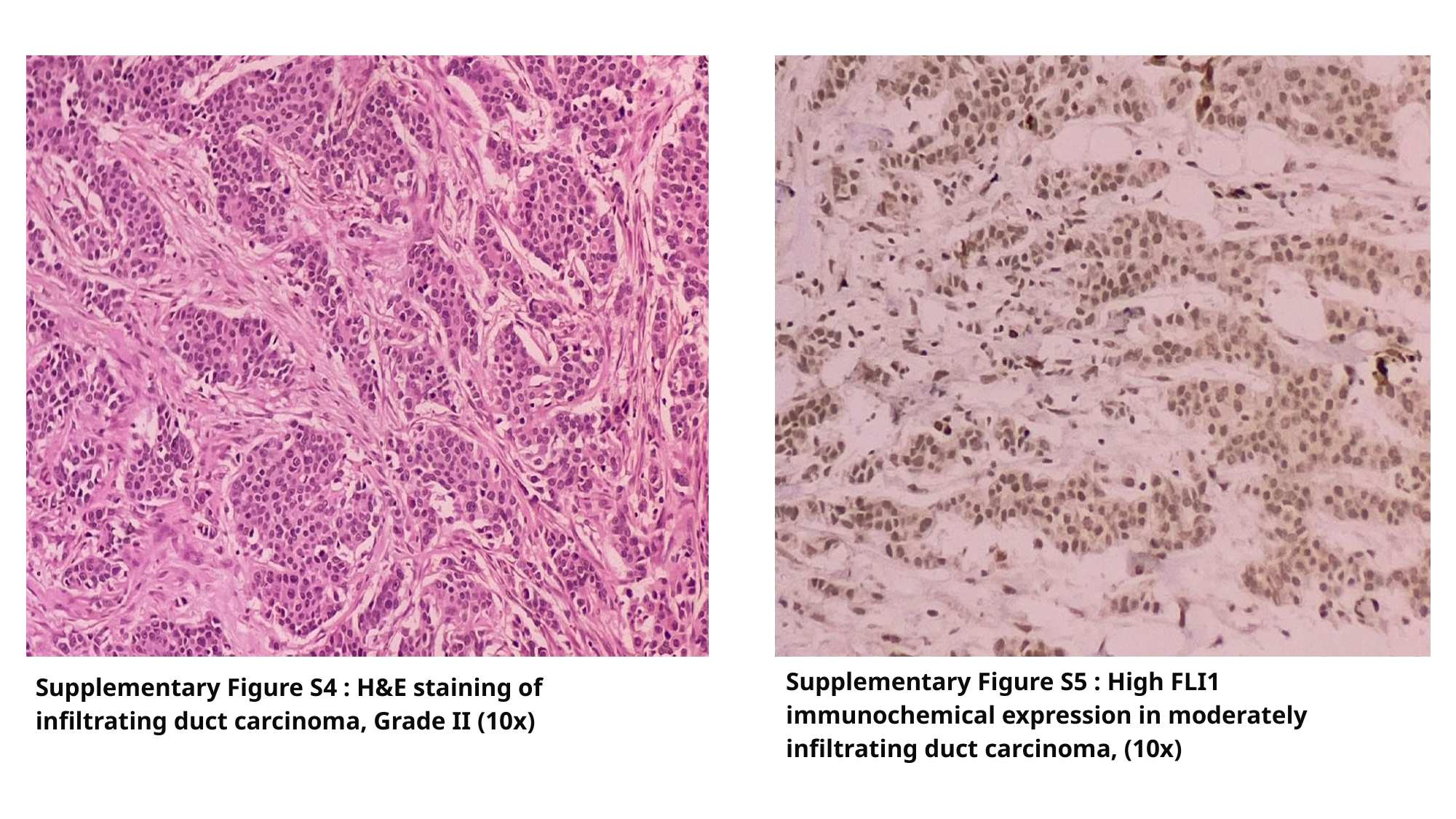

Supplementary Figure S5 : High FLI1 immunochemical expression in moderately infiltrating duct carcinoma, (10x)
Supplementary Figure S4 : H&E staining of infiltrating duct carcinoma, Grade II (10x)

## Slide 4
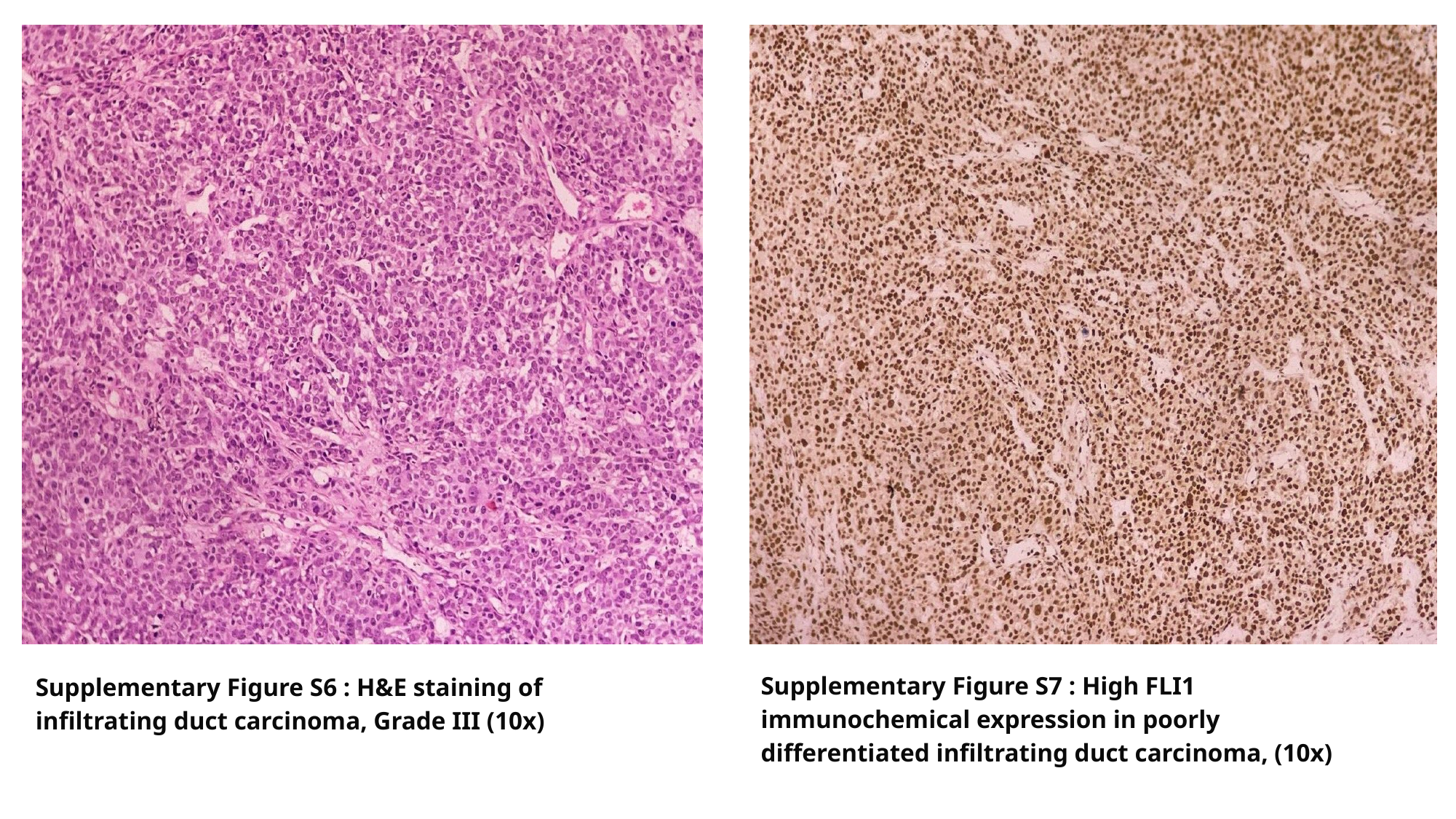

Supplementary Figure S7 : High FLI1 immunochemical expression in poorly differentiated infiltrating duct carcinoma, (10x)
Supplementary Figure S6 : H&E staining of infiltrating duct carcinoma, Grade III (10x)

## Slide 5
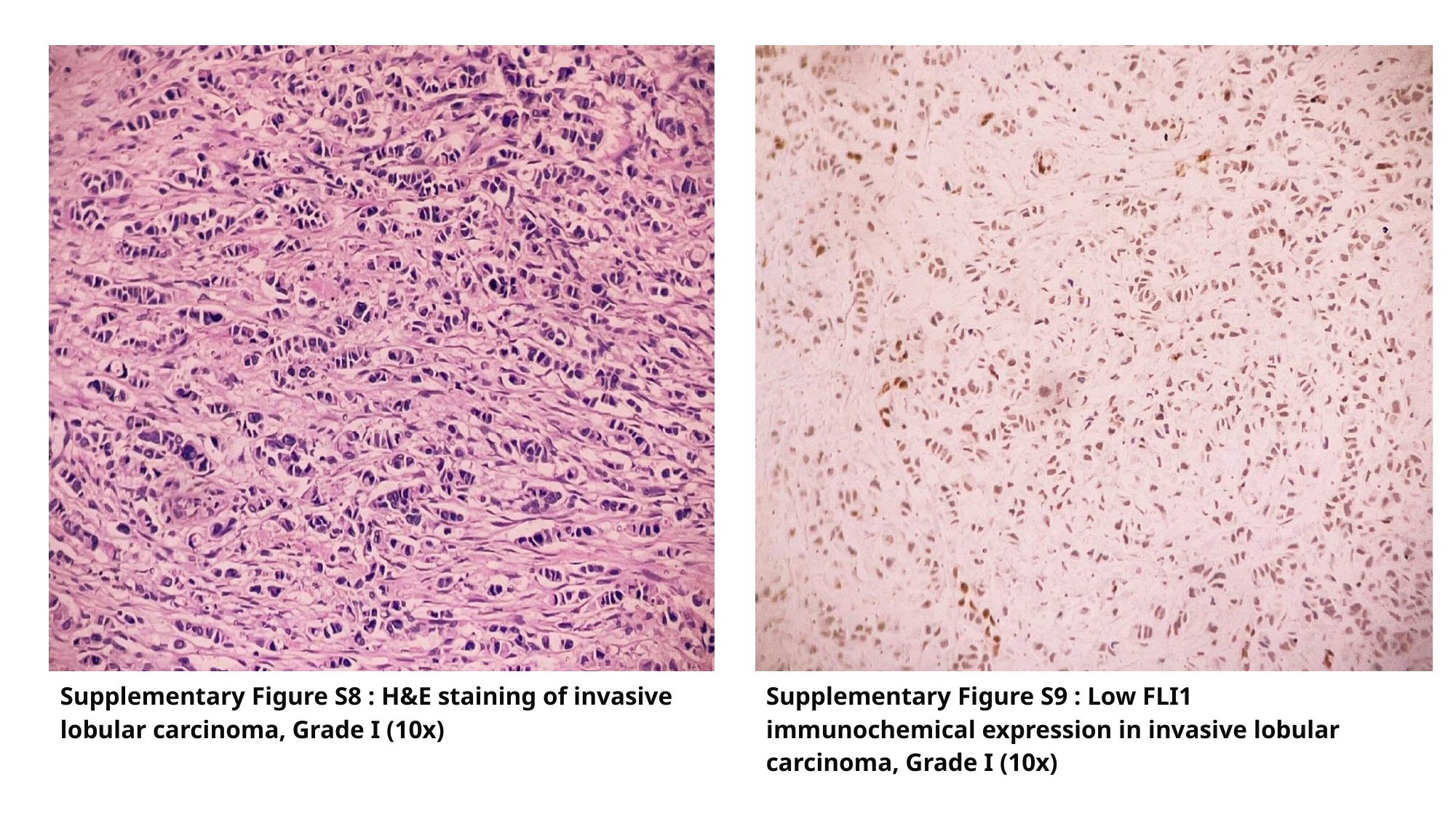

Supplementary Figure S8 : H&E staining of invasive lobular carcinoma, Grade I (10x)
Supplementary Figure S9 : Low FLI1 immunochemical expression in invasive lobular carcinoma, Grade I (10x)
